# Xanthoma Disseminatum Presenting with Hoarseness 

**Published:** 2017-11

**Authors:** Biswanath Behera, Munisamy Malathi, Devinder-Mohan Thappa, Hemanth Vamanshankar, Pradipta-Kumar Parida, Debasis Gochhait

**Affiliations:** 1 *Department of Dermatology, Venereology and Leprology, JIPMER, Puducherry, India.*; 2 *Department of ENT, JIPMER, Puducherry, India.*; 3 *Department of Pathology, JIPMER, Puducherry, India.*

**Keywords:** Hoarseness, Vocal cord, Xanthoma

## Abstract

**Introduction:**

Xanthoma disseminatum (XD) is a rare, benign, non-Langerhans cell histiocytic disorder with unknown etio-pathology. It manifests with multiple, grouped, red-brown to yellow papules and nodules involving the skin, mucous membranes, and internal organs with a predilection for flexures and the face.

**Case Report:**

We report a patient who presented with disseminated xanthomatous papules and nodules involving the face, neck, trunk, axilla, groin, and oral cavity, along with hoarseness of voice. Video laryngoscopy revealed multiple yellowish nodules over the base of the tongue, vallecula, laryngeal surface of the epiglottis, ary-epiglottic folds, interarytenoid region, and subglottic region. Histopathology was suggestive of xanthoma disseminatum and the patient was treated with tablet acitretin 25mg daily for three months without any response. Following this, the patient was prescribed tablet thalidomide 100 mg daily without any significant improvement at the end of two months.

**Conclusion:**

Xanthoma disseminatum is a very rare form of non-Langerhans cell histiocytosis that classically presents with cutaneous xanthomas, mucosal xanthomas, and diabetes insipidus. Hoarseness of voice due to lesions involving the larynx is a rare symptom. Because the disease has punctated, numerous relapses and causes morbidity to the patient, its multisystem manifestations have to be known. Therefore, xanthoma disseminatum has to be kept in mind as a differential diagnosis for hoarseness of voice.

## Introduction

Xanthoma disseminatum (XD) is a rare, normo-lipemic muco-cutaneous xanthomatosis due to benign proliferation of non-Langerhans cell histiocytes. Besides the skin, xanthomatous lesions also affect oral and respiratory mucous membranes, nervous system, and rarely ocular structures and gastrointestinal tracts. We are reporting a case of XD, which presented with hoarseness of voice due to laryngeal involvement.

## Case Reports

**Fig 1:**
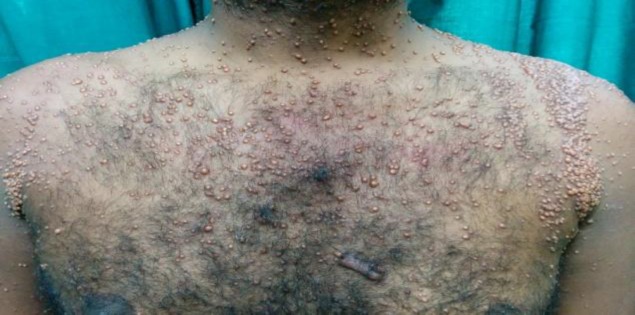
Multiple discrete yellow to reddish-brown papules and nodules present all over the anterior trunk with lesions over the bilateral shoulder coalescing to form plaques and giving a band like appearance.

**Fig 2:**
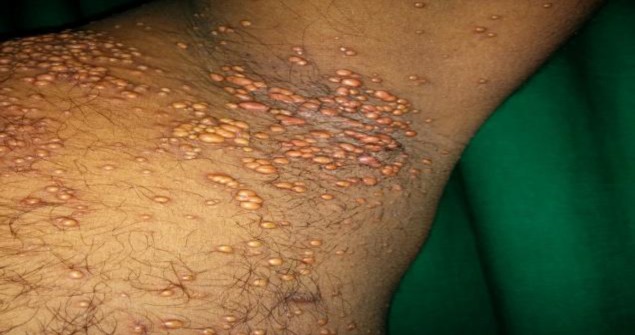
Multiple yellow to reddish-brown papules and nodules grouped together over the left axilla.

A 35-year-old male came to our clinic with numerous, yellow to reddish-brown elevated lesions all over his body for the last three months and with hoarseness of voice for the last two months. Lesions appeared insidiously, initially over the axilla and gradually involved the shoulder, face, scalp, groin and genitalia. There was no history of smoking or hypothyroidism. His family history was noncontributory. There was no history suggestive of nervous system involvment. Cutaneous examination revealed multiple discrete yellow to reddish-brown papules and nodules all over the body. The lesions over flexures and bilateral shoulders were grouped and coalescing to form plaques (Fig1,2).

**Fig 3:**
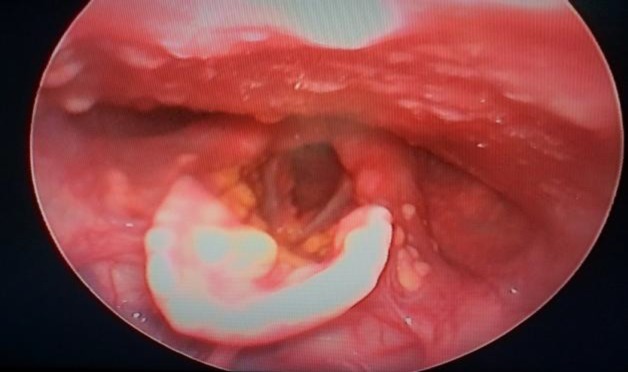
Multiple yellow to reddish-yellow papules and nodules present over the laryngeal surface of the epiglottis, bilateral ary-epiglottic folds, interarytenoid region, and subglottic region.

Examination of the oral cavity revealed multiple yellowish papules and nodules over the buccal mucosa, gingiva, and palate. Video laryngoscopy was performed in view of hoarseness which showed multiple yellowish nodules over the base of the tongue, vallecula, laryngeal surface of the epiglottis, ary-epiglottic folds, interarytenoid region, and subglottic region (Fig. 3). 

**Fig 4:**
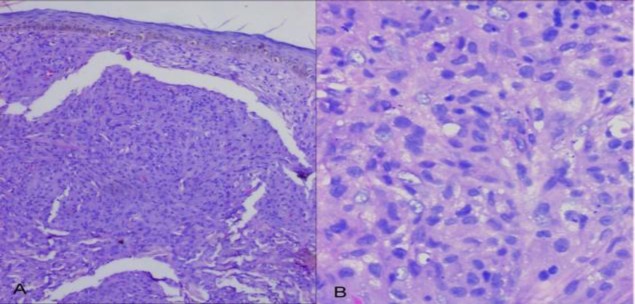
A, Histopathological picture of skin biopsy specimen demonstrating diffuse dermal infiltration by histiocytes (H & E, 100 X). B, Higher magnifications demonstrating both histiocytes few of which are xanthomatized (H & E, 400X)

Both vocal cords were found to be mobile. Ocular examination was within normal limits. Liver and renal function tests, fasting lipid profile (serum triglycerides, total cholesterol, and high density lipoprotein cholesterol), complete hemogram, thyroid function tests, serum electrophoresis, urinary Bence Jones protein, and erythrocyte sedimentation rate were within normal limits. Abdomino-pelvic ultrasonography, chest X-ray, upper and lower gastrointestinal endoscopy revealed no abnormality. Histopathological examination of a cutaneous nodule revealed diffuse dermal infiltration by histiocytes, some of which were xanthomatized, with occasional touton giant cells (Fig 4). 

Immunohistochemical staining revelaed that the histiocytes were negative for S100 and CD1a proteins and positive for CD68 proteins. Following the basis for clinical and histopathological criteria, the diagnosis of XD was reached. The patient was treated with tablet acitretin 25 daily for three months. Due to the patient’s lack of response to acitretin, he was prescribed tablet thalidomide 100 mg daily after base line SNAP (sensory nerve action potential), prothrombin time (PT), and international normalized ratio (INR) testing. However there was no response to thalidomide after two months.

## Discussion

Montogomery’s syndrome or XD is a rare non-familial normolipemic non-Langerhans cell histiocytosis. It manifests as multiple symmetrical xanthomatous lesions with a flexural predilection and is often refractory to treatment. It was recognized as a separate disease entity in 1938 (1). Males between the ages of 5 and 25 are mainly (around 60% cases) affected; however, XD has been described in both sexes and in all age groups (2). 

The classical triad of XD is cutaneous xanthomas, mucosal xanthomas, and diabetes insipidus (3). Three clinical patterns of XD have been described: a common persistent form, a less common progressive form with systemic involvement, and a rare spontaneous regression form (4). Cutaneous lesions are characterized by widely disseminated but often closely set and even coalescing, round to oval, orange or yellow-brown papules and nodules. The lesions have the predilection for flexures and the face. These are found mainly over the neck, axillae, antecubital fossae, groin, and perianal region. Lesions are commonly noted around the eyes (5). In this case, discrete yellow to reddish-brown papules and nodules all over the body with grouped lesions over flexures were observed. In XD, the mucous membranes are affected in 40% to 60% of cases. The upper respiratory tract is more commonly involved compared to the lower respiratory tract and may lead to dyspnea and dysphagia. Rarely, emergency tracheostomy may be required in the case of stridor. Lower respiratory tract involvement is very rare and is generally described during postmortem examination. In our case, involvement of the oral cavity and almost the entire upper respiratory tract mucosa was seen. There was no history of dyspnea or stridor. Meningeal involvement with infiltration of the skull base may lead to diabetes insipidus in 40% of cases, seizures, or growth retardation. Diabetes insipidus is transitory and mild, unlike that seen in Langerhans cell disease. Involvement of the CNS other than the pituitary/hypothalamus region is extremely rare (6). Rarely, hepatic involvement, osteolytic bone lesions, and synovitis have been described. In our case, there was no involvement of the central nervous system (6). We consider our case as an example of a progressive form of XD as old lesions were persistent and new lesions kept arising with the involvement of the entire upper respiratory tract.

The exact etiology of XD is unknown. It is thought to occur due to the reactive proliferation of histiocytes with a secondary accumulation of lipids. However, it is not associated with hyperlipidemia (5). In our case, the serum lipid profile was normal. The foamy-appearing macrophages were believed to be caused by increased uptake, synthesis, or decreased efflux of lipids and thought to be triggered by a superantigen (7,8). 

Histopathology of early lesions predominantly consists of scalloped macrophages while the advanced lesions contain a mixture of scalloped cells, foamy cells, and inflammatory cells, as well as touton giant cells and foreign-body giant cells. XD histiocytes stain positive for lysozyme and alfa one-antitrypsin and also express CD68, CDllb, CD14, CDllc, and factor XIIIa; but are negative for S100 and CD1a (5). In our case, dermal histiocytes were positive for CD68 and negative for S100 and CD1a.

The differential diagnosis of XD includes generalized eruptive histiocytosis (GEH) and progressive nodular histiocytosis (PNH). In GEH, multiple cutaneous lesions appear in crops, generally sparing the flexures. The lesions rarely involve the mucosa and other visceral organs and resolve spontaneously. PNH is characterized by multiple lesions, which progress to form large nodules in older patients, with no evidence of spontaneous regression, and is histologically comprised of spindle-shaped histiocytes (5). 

Various treatment modalities have been attempted but the response to any form of treatment in XD does not yield satisfying results. Surgical excision or laser therapy found to improve physical and functional appearance, but the course of the disease is characteristically punctuated with frequent relapses. 

Various modalities like vasopressin, corticosteroids, chlorambucil, cyclophos-phamide, azathioprine, vinblastine, clofibrate, radiotherapy, and cryotherapy have been attempted with variable response (9). Khezri et al. (9) treated five XD patients with 2-chlorodeoxyadenosine and followed up for three months to eight years, during which no new lesions appeared and all patients had substantial improvement. Our patient was treated with acitretin and thalidomide, without any significant improvement. 

## Conclusion

Xanthoma disseminatum is a very rare form of non-Langerhans cell histiocytosis that classically presents with cutaneous xanthomas, mucosal xanthomas, and diabetes insipidus. XD presenting with hoarseness of voice due to lesions involving the larynx is rare. Because the course of the disease has punctated and numerous relapses and causes morbidity to the patient, its multisystem manifestations have to be known. XD as a differential for hoarseness of voice has to be kept in mind.
